# Extract of the Report of the Foreign Relations Committee of the National Association of Dental Faculties

**Published:** 1900-09-15

**Authors:** 


### EXTRACT OF THE REPORT OF THE FOREIGN RELATIONS COMMITTEE OF THE NATIONAL ASSOCIATION OF DENTAL FACULTIES.

Adopted at Old Point Comfort, Va., July 14, 1900.

During the past year the work of the Foreign Relations Committee has been materially extended. Advisory boards in most foreign countries have been provided. Pamphlets containing an exposition of the work and the aims of the National Association of Dental Faculties have been printed and circulated in foreign countries, and a number of circulars of information for members of our foreign advisory boards have been printed and mailed to them. In addition, as directed by the Association at its last meeting, a pamphlet containing digests of the reports made at that meeting has been printed and mailed to each member of the Association and to other interested members of the profession in America and abroad.

It is unfortunately the fact that, because of the lack of uniformity in the educational systems of the different States, and the absence of any general supervisory authority on the part of the national government, under some unwise local legislation it has been possible for irresponsible, unqualified and unscrupulous men to secure charters for institutions empowered to grant degrees, and under such authority to issue, for a consideration, irregular and fraudulent diplomas. This traffic has principally been with men in foreign countries who, primarily the guilty ones, have sought to attain academic honors without the labor necessary honestly to acquire them. As these institutions have been conducted under pretentious names, it was formerly impossible for foreigners who had no intimate acquaintance with American educational affairs to distinguish between the regular and the irregular schools. The organization of this Association has established a criterion by which they may be judged, only those owning allegiance to the National Association of Dental Faculties being recognized.

It is unfortunate that the professional situation in America has not in past years been better comprehended in Europe. All our schools have been held responsible for the vile work of the fraudulent ones—nominally located in this country, but chiefly supported by unprofessional men from abroad. There has even been a grave misapprehension of the objects of this Association, and the work of the Foreign Relations Committee has in some instances been totally misconstrued. All of us are aware that while some of the very best and ablest American representatives have located in foreign countries, and to whose professional careers we can point with pride, it is unfortunately the case that some Americans of a different professional reputation have gone abroad and have indulged in practices as offensive to our foreign *confreres* as they are to reputable American practitioners. There are many more unworthy foreigners who have legitimately or illegitimately become possessed of an American degree, and who, without warrant or right claim the title of "American dentist."

The belief is prevalent in certain foreign professional circles that it is the aim of the National Association and its Foreign Relations Committee to obtain for all such persons professional recognition and to demand the acceptance of their American degree by the governments of foreign countries. It is but proper that we should in the most authoratative manner deny any aspirations of the kind. This Association has not in the remotest manner contemplated any interference with or protest against the laws or regulations governing the practice of dentistry in any foreign country. It has not primarily been the object of either the National Association or its Foreign Relations Committee to attempt to secure for the American dental degree any legal recognition as a qualification for foreign practice. It is not usual in the American States which have legal professional regulations to receive the diplomas of any foreign professional school as a qualification for practice, and we can not consistently ask that which we refuse to others.

It seems but proper that we should publicly avow the reasons that have prompted the better colleges to form this association of schools, and to appoint a committee charged with the duty of harmonizing our relations with the dental profession in other lands. We seek for the distinctive American dental diploma nothing more than the consideration which its merits demand. If its reputation has been debased by the circulation of counterfeit diplomas, it is something for which we are in no way responsible. Realizing this, the National Association of Dental Faculties was organized for, and has been constantly laboring to attain, these definite purposes:

1. To establish a broad and generally accepted curriculum of dental study, and by the combination of all the better dental schools of America to bring each up to a uniform standard of excellence.

2. To establish a clear line of demarkation between the regular and irregular schools, and to force out of existence the latter.

3. Gradually to raise the standard of preliminary education until none but such as have the general erudition as should distinguish professional men can be accepted in American dental colleges.

These were the principal objects in view, and in the attainment of them success has been secured exceeding the most sanguine expectations of the founders of the movement.

In establishing the preliminary qualifications for matriculation in American colleges there was no rule by which to judge of the value of certificates presented by foreign students. After completing the course of some foreign school a student, who perhaps spoke only a strange language, sometimes desired to conclude his studies by taking as much of the American course as would enable him to finish it, and he demanded of some American college advanced standing of one or more years. His certificates were in a foreign tongue, and in some instances were found either forged or not that which they were represented to be.

In this emergency, at the earnest request of certain American dentists practicing in foreign countries, who had been scandalized by the acceptance in America of students with improper certificates, a committee, to be called the "Committee on Foreign Relations," was appointed, and was charged with certain definite duties:

1. It was to be in all things subordinate and subservient to the National Association of Dental Faculties, to which body it must make a full report each year.

2. It was empowered to appoint advisory boards of not more than three members in each foreign country.

3. It was to have jurisdiction in all foreign educational questions affecting American dental colleges.

4. It was to obtain definite information concerning dental regulations and laws in foreign countries, with the view of determining what value should, under American laws and regulations, be given their certificates of study, either as a qualification for dental practice in America or for admission to advanced standing in American dental colleges.

5. It was charged with the duty of ferreting out institutions engaged in the granting of irregular degrees or degrees irregularly, and instituting measures for their suppression.

Your committee begs to report that it has divided the various countries into convenient groups, and has appointed boards for each, so far as the information obtainable has warranted.

At the very outset colleges, members of this Association, appealed to us to know what consideration should be given to certificates showing that proposed students had taken the full course in schools located in Japan and Mexico, which purported to teach the whole dental curriculum. Your committee could not learn that any schools giving a course in dentistry that could be accepted as an equivalent for any part of that demanded by this Association existed in either country. They therefore ruled that students from either could only be accepted as members of the freshman class of American dental colleges, and only then if they complied with the rules of the Association so far as preliminary education and a knowledge of the English language are concerned. This ruling was cheerfully accepted by the schools that had raised the question, and we present it as an encouraging proof of the loyalty and anxious desire for a high standard that exists among the recognized dental colleges of America.

But the discussion of this raised the question of the consideration that should be given to the certificates of study from any foreign dental school. Our rules provide that no credit shall be given to certificates from any American dental school whose curriculum and regulations have not received the formal approval of this Association. Could we, in the name of the National Association of Dental Faculties, approve the giving of advanced standing to students from the schools of other countries that had not the same stamp of regularity? It took but a short time to arrive at the inevitable conclusion that we could not approve the giving of advanced standing to graduates or undergraduates of any foreign dental school whatever until such school had received the formal indorsement of this body.

It is probable that more fraudulent diplomas have been sold in foreign countries during the past year than ever before. This is due to the fact that those who have been carrying on the traffic realize that the time for accountability is near at hand, and they are striving to make the most of the present opportunity.

It is urged by foreigners that this business should be summarily stopped. They little know the difficulties in the way. In the first place, the traffic is mostly with foreigners As their illegitimate diplomas are wholly worthless in this country, no State Board of Examiners recognizing them in any way, those who are engaged in the business carefully cover their tracks, and no responsible man can be located. Attempts to entrap them by means of decoy letters have failed, some such having crossed the ocean a number of times without delivery, being forwarded from one of their foreign agents through whom the nefarious business is carried on to another, until finally returned to the writer by the post office authorities Fictitious names are signed to the pretended diplomas, so that it has been found almost impossible to fix the guilt upon any person Our friends in foreign countries have contented themselves with bitter reproaches against American colleges generally, without forwarding any testimony that would assist in the discovery of the guilty ones The fraudulent institutions can not be distinguished from the regular colleges by foreigners for they are in possession of charters regularly granted under a vicious law of the State of Illinois.

Your committee early discovered that working alone it could accomplish little. The Board of Health of the State of Illinois was taking the matter up, and it possessed advantages for the prosecution of the lawbreakers which were not within our reach We have therefore contented ourselves with co-operating with that board in every way possible, and our counsel has been instructed to offer any assistance within his power. As a consequence we have great pleasure in reporting that, acting under the United States law, which forbids the use of the mails for fraudulent purposes, the worst of these offenders have finally been apprehended and committed to jail in default of the heavy bail that was demanded. What is of more importance, the United States mails are closed against the transmission of their correspondence, and letters to or from them are promptly sequestrated.

The greatest offender was last year named in this report as "The Independent Medical College of Chicago." We secured the annulment of the charter of this affair, but in a very short time we found that the same men were yet engaged in the business under the name of "The Cosmopolitan Medical College." They had offered for sale no less than thirty-six different diplomas in all the branches of science and art, and since the forfeiture of the charter under which they first worked it is believed they have sold more than a thousand fraudulent diplomas, at prices varying from $10 to $500 each. Proof sufficient to secure the cancellation of the first charter was only obtained through the inordinate cupidity of the man who was chiefly responsible. He paid a debt of some $30 due to a stable man, or hostler, by issuing a diploma to him and making him a professional man The recipient, when he found himself under arrest for attempting practice under it, betrayed the swindler, and we were thus able to fix his guilt.

The late proceedings against this man and his associates have developed the fact that they were in possession of no less than *twenty-four* different charters, all regularly issued under that mischievous Illinois law. which was enacted for beneficent purposes. We have now learned the methods of these men, and it is believed that it will soon be possible to put an entire stop to their villainous traffic, through the imprisonment under the United States postal laws of those engaged in it. Too much credit can not be given the Board of Health of the State of Illinois for the active part it has taken in the suppression of these miserable pretenders that have so long been bringing discredit upon our legitimate and excellent educational institutions.

The progress that this Association is making in its efforts to raise the status of professional teaching in our own country, to obtain a better appreciation of American professional affairs in foreign countries, and to maintain steady advancement toward a dental solidarity among all nations is very encouraging to every lover of humanity. It is true that even at home there may in uninformed circles yet be found some remnants of an unworthy professional jealousy, a failure to comprehend the real educational situation, and a tendency to attribute to our teachers motives unworthy any honest man But the steady, persistent work of this Association in elevating the accepted standard just as fast as prudence permits, has wrought a great change in professional sentiment and immeasurably benefited the schools, and through them the profession at large. It only remains for us to continue this good work a few years longer to produce results that will be permanent in their character, and so firmly established as henceforth to be self-sustaining.

### REPORT CONCERNING FOREIGN EQUIVALENTS.

It must not be forgotten that the system of dental instruction in Europe varies very widely from that of our special American dental schools. Instruction separate from that given in the medical schools or universities is very rare, and the practical training which forms a part of our curriculum is usually given by private preceptors.

Your committee does not feel at liberty to recommend the acceptance of an oral and theoretical course as the equivalent for one including practical work. We can not believe that the certificates of private and irresponsible practitioners can by us be accepted as any part of a college course, and hence we have given them little consideration.

*Students or graduates of foreign schools can be given advanced standing only in conformity with the following rules:*

1. The college must require of matriculants a preliminary education which is the full equivalent of that demanded by the schools of this association.

2. The college must demand of students full attendance upon at least three full annual courses (not semesters) of lectures of not less than seven calendar months each, in separate years, covering all the studies proper to a full dental curriculum.

3. The college must possess a bacteriological laboratory, with sufficient of equipment for instruction in a competent course in bacteriology, which must form a part of its curriculum of s'udy.

4. The same must be required in chemistry, his'ology and pathology.

5. There must be a technic laboratory in which shall be taught the proper manipulations for the insertion of all kinds of fillings for teeth, the preparation and filling of the roots of teeth, the tempering and shaping of instruments, the drawing of wire and tubing for cases in orthodontia, and the cutting of bolts and nuts.

6. There must be prosthetic laboratories sufficiently equipped for teaching all kinds of prosthetic work and the construction of all the approved prosthetic appliances.

7. There must be a sufficiently equipped laboratory for instruction in making crowns and bridges, and the construction of appliances used in orthodontia.

8. There must be a properly equipped infirmary or surgery for the reception of patients, upon whom each and every student shall be required individually to perform all and enough of the operations necessary in dental practice thoroughly to qualify him for the successful pursuance of his profession.

9. Complete records of the work done by each student, of his attainments at sufficient and full examination in each subject of the curriculum of study, of his attendance and deportment during the course, must be permanetly kept.

10. No credit must be allowed for any work not done under the immediate supervision of instructors connected with or especially approved by the college, and who are in direct affiliation with the faculty.—*Cosmos.*

